# Learning to teach in medical education

**DOI:** 10.3205/zma001535

**Published:** 2022-02-15

**Authors:** Marjo Wijnen-Meijer

**Affiliations:** 1 Technical University of Munich, School of Medicine, TUM Medical Education Center, Munich, Germany

## Editorial

Teacher professionalization takes on a significant role in medical education, because only competent teachers can make education excellent [1], [2]. Especially, in higher education curricula changes regularly to address the expanded scope of knowledge, educational technologies, and active teaching approaches [3]. Therefore, it is necessary that faculty teachers have appropriate skills and qualifications for teaching [2]. 

The study by Blitz et al. [4] in particular shows how students think about teaching. They describe tensions between students' expectations of faculty and their experiences of clinical teaching. Students feel that teaching was secondary to patient care. In medical education, it is important that students' expectations of teaching are met. They need support in developing their skills as well as in their personal way of consuming knowledge and developing their ability to act. This requires an understanding of faculty development and the strengthening of clinical teaching [4].

Faculty development includes all activities that improve teachers' knowledge, skills, and behaviors [1]. Specifically, this means that teachers learn how to put their knowledge into practice to help their students develop. For teachers, professional learning is a complex process that requires cognitive and emotional engagement to consider and implement appropriate alternatives to improve or change teaching practices [5]. In this process, certain educational policy environments or school cultures influence, on the one hand, the goals and needs of students and teachers. On the other hand, the instruments used for improvement, such as courses or workshops are having an impact on teaching and learning behaviors [5]. In response, most medical schools offer a variety of programs and activities with different format and purpose to help faculty improve their skills as teachers and educators [1]. The different interventions described in studies were workshops, short courses, seminar series, longitudinal programs, other activities such as peer observations and web-based modules. The program effectiveness is contributed to key features like evidence-informed educational design (use of multiple instructional methods), relevant content, experimental learning and opportunities for practice and application, opportunities for feedback and reflection, educational projects, intentional community building, longitudinal program design, and institutional support [1]. 

The center of the professional development are the teachers, they continue to be both the subjects and objects of learning and development [5]. They can develop their teaching expertise through mentoring by medical educators, collaboration between academics and colleagues, interaction with students, online learning, workplace learning, and use of learning technologies [1], [3], [5], [6]. Dutt et al. [3] highlight ways in which professional educators can develop their teaching and what influences their development as teachers. Especially important is feedback and time for reflection and self-monitoring in the continuous professional development. In the process some teachers changed from the lecture style into an active learning approach in which students are more integrated [3]. 

Based on different educational theories, Dennick [7] has collected 12 tips for this active learning approach, which are particularly useful for healthcare teacher.

1. It is important to identify and activate students' existing knowledge. It allows the teacher to build a relationship with the students, by demonstrating empathy and respect.
2. Existing knowledge can be built upon. Not only should cognitive connections be made to new learning content, but the significance and relevance should be emphasized. The aim is to question the existing knowledge in order to elaborate or refine it.
3. Use the social context of learning in group work to understand new terminology and concepts.
4. Effective learning comes from active learning techniques, by applying knowledge and problem solving.
5. Students should self-reflect and think about how they learn. This makes the students responsible for their own learning.
6. Students should get the experience they need either individually or as a part of a group. The experiences could be through social interaction, activity and discussion.
7. The experiences are transformed into learning through reflection, which happens either consciously or unconsciously. This process can be supported by other individuals or by writing in for example log-books or portfolios.
8. When reflecting on experiences, it is important to consider practical skills and attitudes.
9. Students need to be given the opportunity to apply what they have learned, for this they may need support from facilitators or mentors.
10. Within the curriculum, students should be able to pursue their own interests.
11. To create a good learning environment, the physiological and psychological needs of students must be satisfied. This means, for example, that learning spaces are a pleasant environment and positive feedback should reinforce self-esteem, self-efficacy and self-actualization.
12. Last, learning is characterized by the relationship between the learner and the teacher. Students should be members of curriculum and teaching committees [7].

Nevertheless, the active learning approach is challenging especially in the interaction with distance students via video-conference and the teachers are concerned that content may not be completely covered in preparatory material and face-to-face teaching [3]. The COVID-19 pandemic represented a unique form of restriction on medical education in the in-person teaching. To ensure the safety requirements a rapid change from conventional teaching methods to virtual formats was needed. It was essential that during this time teachers adapted to the circumstances and changed their teaching style in order to ensure that medical education could continue [8]. Virant-Young et al. [8] describe a live regional hub course for all teachers. The content included a simulated walkthrough of all components of the virtual course, instruction on balancing workshop content with small group, breakout activities and the use of the virtual platform. It is necessary to counteract the fear that virtual education is less effective than face-to-face teaching. Therefore, it is essential to show teachers in the teaching programs that a virtual platform can be designed to bridge the gap between just viewing a program on a screen to actively interacting with other participants and instructors [8]. This is just an example of how faculty development can quickly adapt to newly occurring circumstances.

Other existing challenges are the lack of clarity and visibility of the faculty teacher´s role. Many of the university teachers are engaged in clinical or research activities and view teaching as an add-on activity [2], [4]. Some teacher acquire their didactical knowledge mostly through experience, experimentation, and feedback from students without attending faculty development programs [1], [2], [3]. Resistance to teacher professionalization programs is not due to a lack of interest on the teacher's part, but rather due to the time commitment, lack of resources, and rewards [2].

A model that outlines the conditions for change and teacher development is the 4-C framework, described by Van Bruggen et al. [2]. According to this, faculty development depends on 4 factors.

1. Competence, which teachers need in order to perform their teaching duties.
2. Context, this refers to the resources teachers require not only to fulfill their teaching duties but also to develop and enhance personal skills.
3. Community of practice, to support, collaboration, mentoring and for advocacy.
4. Career, means the visibility of the career as a teacher. All 4 factors are necessary for faculty development and institutional change. If one component is disadvantaged, the effectiveness of the program is reduced [2].

A comprehensive approach to faculty development that addresses teachers' individual career goals related to professional development and growth is presented by Bailey et al. [9]. For this purpose, a diverse set of offerings is provided that includes 5 content themes: teach, leadership, discover (scholarship), advances (policies, engagement, ...), and service. These 5 topics are sub-categorized by career stage to allow teachers to identify their competencies with the career goal. As a result of this expanded offering, 35 percent more participants are attending the courses. In addition to providing support for teacher development, the new faculty development structure allows improvement in planning and reporting of faculty development activities [9]. However, this development indicates, not one form of professional development is relevant to all teachers [5]. It may be time to re-conceptualize faculty development. By understanding faculty development as an opportunity for renewal and reflection on personal and professional growth, rather than solely for the enhancement of professional skills [1], [9].

It is important that the content of the teacher professionalization offer is constantly adapted to developments in the curriculum and new didactic insights. This issue discusses a number of topics that may deserve a place in teacher training programs. Schrempf et al. [10] describe the relevance of mentoring and guiding students in reflecting on their competence development. It may also be relevant if teachers recognise when students are using intuitive concepts, as described in the article by Harendza & Herzog [11]. Another possible topic is learning to recognise depressive symptoms among students, so that they can be offered help quickly. As Pukas et al. describe, this is a common problem [12]. Finally, teaching research skills, for example in the form of journal clubs for medical students as proposed by Taverna et al., can also be a topic of faculty training [13].

## Competing interests

The author declares that she has no competing interests.
